# What Fuels the Fire: A Narrative Review of the Role Social Determinants of Health Play in Burn Injuries

**DOI:** 10.3390/ebj3020033

**Published:** 2022-06-16

**Authors:** Kimberly H. Khoo, Emily S. Ross, Joshua S. Yoon, Tomer Lagziel, Feras Shamoun, Joseph S. Puthumana, Julie A. Caffrey, Sheera F. Lerman, Charles Scott Hultman

**Affiliations:** 1 Department of Plastic and Reconstructive Surgery, Johns Hopkins University School of Medicine, Baltimore, MD 21205, USA; eross26@gmail.com (E.S.R.); jyoon54@jhmi.edu (J.S.Y.); tlagziel1@jhmi.edu (T.L.); fshamou1@jhmi.edu (F.S.); jputhum2@jhmi.edu (J.S.P.); jcaffre5@jhmi.edu (J.A.C.); chultma1@jhmi.edu (C.S.H.); 2 Department of Psychiatry, Johns Hopkins University School of Medicine, Baltimore, MD 21205, USA; szohar1@jhmi.edu

**Keywords:** burn, social determinants of health, economic stability, education access, health care access, neighborhood and built environment, social and community context

## Abstract

Social determinants of health (SDOH) are the conditions where people live, learn, work, and play that affect their health and quality of life. There has been an increasing focus on the SDOH in the field of medicine to both explain and address health outcomes. Both the risk of burn injuries and outcomes after burns have been found to be associated with multiple aspects of the SDOH. This narrative review seeks to explore the main domains of the social determinants of health, reiterate their importance to the general and burn injury population, examine each’s association with risks of burn injuries and burn-related outcomes, and provide an overview of the current burn research landscape that describes the social determinants of health.

## 1. Introduction

The social determinants of health are “conditions in the places where people live, learn, work, and play that affect a wide range of health and quality-of life-risks and outcomes.” [1] Though broad, this definition encapsulates the overarching understanding that medical care alone is not sufficient to explain and promote a person’s health status, likely only explaining 10–15% of preventable mortality in the United States [2].

In the public health, sociology, and behavioral sciences fields, the term “social determinants of health” (SDOH) arose after decades of evidence proving that health is influenced by more than just the medical care a patient receives. The field of medicine has recently begun to adopt and highlight this term in research and practice, with efforts pushing for education about SDOH in medical schools [3,4], interventions built around these social determinants [5], and continuing medical education that teaches healthcare professionals about the importance of these factors [6].

The World Health Organization (WHO) lists examples of various social determinants of health that may positively or negatively influence health and health equity: income and social protection, education, unemployment and job security, working life conditions, food insecurity, housing and basic amenities, early childhood development, social inclusion and non-discrimination, structural conflict, and access to affordable health services of decent quality [4]. Healthy People 2030, a federal agenda for improving health and well-being, groups SDOH into five main domains that encompass those described by the WHO: economic stability, education access and quality, health care access and quality, neighborhood and built environment, and social and community context [7].

There is evidence showing that intervening on specific SDOH may improve health outcomes, such as obesity or chronic disease, and reduce healthcare spending [8]. With the understanding that these factors play a large role in the health and outcomes of patients, it behooves physicians to become familiar with the various SDOH and the pathways along which they impact health. Especially considering that burn injuries are a global public health problem frequently correlated with socioeconomic status [9], burn health care providers should be aware of the different SDOH and how they may impact a patient’s risk of burns and outcomes for recovery. This review seeks to explore the main domains of the social determinants of health, reiterate its importance in the general and burn injury population, examine each’s association with the risk for burn injuries and burn-related outcomes, and provide an overview of the current burn research landscape that describes the social determinants of health.

## 2. The Social Determinants of Health

The five main areas of SDOH are economic stability, healthcare access and quality, education access and quality, social and community context, and the neighborhood and built environment. Each of these domains can be further detailed by specific factors that may go on to affect a patient’s quality of life and health [8].

### 2.1. Economic Stability

Economic stability includes key considerations such as poverty, employment, housing stability, and food security [7]. Especially in developing countries, economic stability plays a crucial role in influencing health, and both developing and developed countries experience a positive correlation between health and income [10]. Economic factors may constrain healthy behaviors, such as eating whole foods or attending annual doctors’ visits, and may promote exposure to unhealthy environments such as high-risk working conditions [11].

### 2.2. Healthcare Access and Quality

The domain of healthcare access and quality includes access to regular services such as preventative visits and cancer screenings. It also covers treatment for drug and alcohol use, health literacy and communication, health technology access, access to health and dental insurance, screening for sensory or communication disorders, and rehabilitation after disability. Literature shows that access to healthcare is a strong predictor of positive health outcomes. A survey of 11 countries found that adults in the United States were more likely than any of the other 10 countries to go without needed health care due to higher costs [12]. Though 91.4% of people had health insurance coverage for all or part of 2020 [13], access issues persist for adults who are adequately insured, with 23% reporting at least one cost-related problem getting health care [14]. Cost is not the only issue though, as many patients lack physical access to their doctor, whether that be due to a shortage of local physicians, the inability to travel for a check-up, or the inability to access technology that may facilitate better health, such as online access to medical records or appointment scheduling [7].

### 2.3. Education Access and Quality

The Healthy People 2030 domain of education access and quality addresses issues such as readiness for school and graduation, increasing the proportion of students with disabilities in regular education programs, and the promotion of basic reading and math skills [7]. Extensive research has been conducted to delineate the relationship that exists between health and education, particularly because education is so interwoven with other social and economic factors such as job stability, health literacy and historically contextual social classes. One landmark study by Kitagawa and Hauser described differences in mortality by education level in the US [15], and other research has found that adults who are less educated have higher rates of chronic conditions, poorer health, and disability [16]. The impact of educational disparities compounds over the years, as children who come from low-income families or who are disabled may not receive adequate educational support, which puts them at a disadvantage throughout the rest of their educational path and may perpetuate the cycle of poverty. 

### 2.4. Social and Community Context

This domain focuses on the importance of relationships and interactions with family, friends, co-workers, and community members as promoters of health and well-being. It includes topics such as the mental health of caregivers of people with disabilities, communication between adolescents or children with their families or a loved one, health communication and health literacy, bullying of transgender students, and the proportion of patients with intellectual and developmental disabilities who live in institutional settings [7]. Social support contributes greatly to how people respond to other negative factors in their lives. In the medical and behavioral sciences, this is recognized by the idea of attachment theory and how forming certain emotional and social bonds during critical timepoints in life is necessary for normal development [17]. When thinking about SDOH and their impact on health, social networks and social support are considered. Social networks are defined as the “web of person-centered social ties” [18], while social support refers to “the various types of assistance people receive from their social networks […] differentiated into three types: instrumental, emotional, and informational support” [19]. Social support and the social context have been shown to promote positive physical and mental health outcomes [20] as well as improved resistance against the development of infections [21], prognoses and chances for survival after a serious illness [22], and health outcomes such as morbidity and mortality [19,22]. 

### 2.5. Neighborhood and the Built Environment

This domain covers objectives such as increasing access to the internet, decreasing rates of youth violence, decreasing the amount of pollution released into the environment or water, decreasing lead levels in housing, reducing motor vehicle accident rates, increasing the proportion of adults walking or biking places, reducing tobacco use, and promoting public transportation [7]. The built environment is defined by the CDC as “all of the physical parts of where we live and work (e.g., homes, buildings, streets, open spaces, and infrastructure)” [23]. These built environments and the neighborhoods in which people live can influence their health through direct impacts, such as unsafe drinking water, high rates of violence in the area, or pollution, the latter of which has been linked to increased deaths from respiratory and cardiopulmonary diseases [24,25]. Built environments can also have indirect influences on health by influencing a person’s health behaviors. For example, in a neighborhood that has fewer sidewalks and trees, people may be less likely to exercise outside or choose to take public transportation. Environments that impede an active lifestyle may contribute to higher rates of obesity and poor nutrition [25,26,27]. 

Why has there suddenly become such an emphasis by the wider medical community on these social determinants of health? Prior focus was on “risk factors” as a means to prevent disease, which heavily emphasizes a patient’s personal choices as the root of unhealthy behaviors. This, however, has proven to be ineffective as many factors lie out of a patient’s hands, such as those listed in the five domains of the social determinants of health [28,29]. With this understanding, and as the US healthcare system has shifted to a more value-based model that rewards outcomes over treatments [30], it has caused physicians and healthcare providers to think more critically about the networks and causes outside of the patient alone that may cause poorer health outcomes.

Additionally, there is increasing emphasis on the promotion of health equity. Defined by Healthy People 2030 as “the attainment of the highest level of health for all people” [31], it stresses that all people should have the opportunity to lead healthy lives. Health equity is deeply intertwined with other ideas such as health disparities, health care disparities, and health inequalities, as neatly delineated by Gomez et al. [32]; however, one of the main concepts to keep in mind with the terms “health equity” or “health inequities” is that health inequities are avoidable, and active work is required to prevent and dismantle health inequities in our communities [33]. To address these health inequities and disparities with the overarching purpose of improving the health of all people, the SDOH must be at the forefront of clinical decision making.

## 3. Social Determinants of Health and Burn Injuries

A major public health crisis, burn injuries are the fourth most common type of trauma worldwide [34,35,36,37,38]. There are established links between socioeconomic factors and burns. When looking at burn injuries through a “risk factor” lens, the risks of burns related to socioeconomic factors include living in crowded housing, low maternal education, and high rates of unemployment [37,38]; studies have even shown that burns occur in poorer socioeconomic areas [39,40]. Moreover, burns, especially childhood burns, are fundamentally preventable, with modifiable risk factors such as lapses of supervision, walking barefoot, the improper storage of flammable materials, a lack of enclosures for open fires for cooking or heat, a lack of water supply, or illiterate parents or caretakers [34,39,41,42,43,44,45,46]. Considering this preventative nature, as well as the published risk factors for burn injuries, it is befitting that burn health care providers be aware of the SDOH and how these factors may place patients at risk for burns or for poorer outcomes after burn injuries.

To gauge the current landscape of knowledge regarding SDOH and burns, we reviewed the current literature that describes each of the five domains of the social determinants of health, as delineated by the Healthy People 2030 initiative [7], in relation to burn injuries and outcomes for burn-related injuries. We conducted an electronic literature search using the PubMed, Embase, and CINAHL databases via access through the Johns Hopkins University library system. No limitations were applied to the search, and a combination of MeSH and non-MeSH terms were used for each database. The search strategies were selected to ensure the inclusion of enough studies that discussed if the specific SDOH domain impacted the risk for burns, mechanisms of burns, access to care, outcomes, and what patient populations get burned (Table 1). Studies included were additionally reviewed for relevant cited articles that may also help to explain a certain SDOH domain’s association with burns.

### 3.1. Overall Literature Regarding Social Determinants of Health and Burns

Few studies exist that examine the relationship between burn injuries and the social determinants of health, using the term as the primary variable. This is likely because these social determinants are multifaceted and broad, which is why subdividing SDOH into domains can provide a more granular understanding of how each factor impacts health.

Regardless, numerous studies have been published trying to understand “social factors”, “social complexities”, or “socioeconomic differences” in relation to burn risks or outcomes. In a comprehensive systematic review, Padalko et al. reviewed 47 studies and found that “social complexity factors” in a child’s environment, such as belonging to a lower income family, having a behavioral disorder, having a parent with fewer years of education, and residing in a rural setting, are associated with an increased risk for burn injuries in children [47]. Based on the results of this review, they conducted a case-control study at a Canadian regional burn center to identify exactly which SDOH influenced burn injuries. Their multivariable model suggested that children from low-income households, in care (removed from the family of origin and placed in the care of another adult due to concerns of neglect), from a family receiving income assistance, or children of teen moms experienced higher odds of having a burn injury [48]. Argirova and Arnautska, in a retrospective review, noted that the children most at risk of burns were those coming from poor and distant regions or raised by single parents who were of lower social status and had low educational levels [49].

Similar studies have been conducted to understand the factors associated with burn injuries in adults. A cross-sectional analysis of burn patients hospitalized in Isfahan, Iran, showed that burn-related mortality is associated with a lower financial status [50]. A retrospective chart review conducted on 878 burn patients showed that drug dependence, homelessness, and the number of emergency department visits were factors associated with missed follow-up appointments [51]. A systematic review by Edelman demonstrated that while the definition of “socioeconomic variables and outcomes” were variable across studies, there were patterns regarding these factors seen in the literature: burn risk is associated with poverty, education, and unemployment; large and single-parent families are at increased risk; housing and crowding play a significant role in the increased risk for burns [38]. In elderly patients, one study noted that increased poverty levels and decreased educational levels were predictors for being discharged from a skilled nursing facility, and that poorer socioeconomic factors were associated with worse outcomes in the patient population [52]. Based off these findings, the article concluded with a call to action:

*“As we care for these older patients, a better understanding of social determinants of health may be useful in making informed medical decisions. Future studies should include data on socioeconomic status and social determinants of health in this age group”.*

In the following sections, we will summarize results from reviewed studies that meet this call to action as they explore the various domains of the SDOH and their respective impacts on burn patients. 

### 3.2. Economic Stability

Economic stability, or rather, the lack thereof, has been shown to be correlated with the risk for burn injuries. One study interviewed children in South Africa to investigate their understanding of the causes of childhood burns. Themes that appeared from the interviews centered around unsafe living structures (lack of electricity, overcrowded housing) or inadequate supervision and compromised caregiving (inebriated parent, parent leaves home after lighting the stove) [53]. A retrospective chart review of patients in the southwest US with injuries to their total body surface area (TBSA) >15% found that socioeconomic status (determined by zip code and US census data) was only associated with increased burn risk in patients who identified as women [54].

Many of the studies considered economic stability as an outcome measure after burn injuries rather than a predictor or factor that may cause burn injuries. Nineteen of the returned articles were qualitative or quantitative studies seeking to determine what factors could predict if, when, and how a burn patient returned to work after an injury and what the barriers were for a patient returning to work. These studies are not included in this review.

Two studies focused on a related concept of “social deprivation”, defined as “limited access to society’s resources due to poverty, discrimination, or other disadvantage” [55]. One study based in New Zealand identified significant health disparities in that the burden of burn injuries was higher in Maori and Pacific Island people, who are more likely to have lower incomes, lower educational attainment, and to live in poor, overcrowded housing [56]. Another retrospective analysis of 450 records in Wrexham, Wales, found a statistically significant association between a patient’s index of multiple deprivation (IMD) score (pertaining to the level of social deprivation based on where a patient lives) and the incidence of burns [57]. These studies support previous research that shows an association between social deprivation and the incidence of fatal fire incidents [58]. Kaufman et al. demonstrated that patients who were victims of burns caused by assault were more likely to be female, black, homeless, unemployed, unmarried, and funded by Medicaid [59]. Sethi et al. conducted a study in Winnipeg, Manitoba, Canada, and found that adults who were in the lowest area-level income quintile in this region had a 5.4 times higher incidence of burns than patients in the highest income quintile [60]. Locke, Burke, and Rossignol, in a series of studies from 1986 to 1990, reaffirmed that poor socioeconomic status results in an increased burn risk for patients at the census level and even demonstrated that certain economic factors, such as employment status, are associated with and can predict the incidence of burn injuries [61,62,63].

### 3.3. Health Care Access and Quality

Most studies analyzing the relationship between health care access and quality and burns focus mainly on two aspects of this domain: health insurance and access to burn centers, which often correlates to the quality of burn care received. With over 40,000 burn injuries requiring hospitalizations [64] and the reported mean total healthcare cost for burn patients in high-income countries being $88,218 [65], burn injuries present a significant economic burden not only to the healthcare system but also, most importantly, to the patient.

A few studies have examined the relationship between health insurance status or primary payer status on health outcomes for burn patients. Farrell et al. found in their prospective study that patients without health insurance were seven times more likely to be discharged home compared to those with insurance [66]. Peluso et al. utilized the Healthcare Cost and Utilization Project (HCUP) National Inpatient Sample (NIS) to demonstrate that Medicaid was associated with higher morbidity and healthcare resource utilization (length of stay, total charges during admission) as compared to patients with no insurance, who were associated with shorter hospital stays and lower total charges and costs [67]. Two studies capitalized on the implementation of the Affordable Care Act (ACA), which expanded Medicaid eligibility to adults whose incomes are up to 133% of the federal poverty level. With the implementation of the ACA, the number of people uninsured has reduced to historically low levels [68], and Dvorak et al. and Oh et al. examined the impact that this reduction had on outcomes of burn patients. Dvorak et al. examined the HCUP-NIS database between the years of 2011 to 2016, with January 2014 set as the intervention time, and noted an association between the expansion of Medicaid eligibility and patient discharge settings, with more patients being discharged to nonacute, healthcare facilities [69]. Oh et al. analyzed the same impact but at a single-center institution, the University of Washington Regional Burn Center. They analyzed patients between 2011–2013 (“before” ACA’s Medicaid expansion) and 2015–2018 (“after”) and found that the average acute length of stay increased. Like Dvorak et al., there was an associated increase in patients being discharged to inpatient rehabilitation centers instead of their home [69]. Both studies noted no difference in mortality before and after the expansion [69,70].

A small number of studies investigate the “health care access” portion of this SDOH domain. The unique care that burn patients require lends itself to inherent issues with access to care, as burn care is resource-intensive and requires expert clinicians spanning multiple healthcare fields. These resources and staff are often located at dedicated burn centers. Centers are qualified and certified to be “burn centers” through a process overseen by the American College of Surgeons (ACS) and the American Burn Association (ABA), and the ABA has published criteria related to burn injuries that may guide a physician’s choice to transfer a burn patient to one of these certified burn centers [71]. Patients with more severe or extensive injuries, especially, should be treated at certified burn centers, and the paucity and distribution of these burn centers in the US contributes to challenges in accessing critical, life-saving burn care [72]. Klein et al. note that physical, geographic access to a certified burn center varies across regions in the US, and that a minority of the population lives within 2 h via ground transport of an ABA-certified burn center [73]. A descriptive analysis analyzed the outcomes of transporting burn patients to a single, regional burn center. They found that the average transport time from injury to the authors’ institution was 7.2 h, though there were no reported patient deaths [74]. Another study found that patients being admitted directly to burn centers were more likely to have experienced inhalational injury, to require intubation, to develop an infectious complication, or to require emergency procedures [75]. A qualitative study by Zadeh et al. sought to identify the challenges associated with delivering burn care in Iran. Through the thematic coding of interviews, the authors found that insufficient access to health professionals, both in terms of the quantity of burn physicians and the availability of existing physicians, led to poorer morbidity or mortality in burn patients. They also noted that poor access to other specialists in specialized burn hospitals (SBHs) and a lack of patient follow up due to financial barriers served as challenges in specialty burn care [76]. The nature of how burn patients are triaged to certified burn centers based on certain criteria presents a challenge to studying the direct impact that access to one of these centers may have on burn outcomes. Additionally, no other studies were found that investigated the association between burn risk and incidence with access to or the quality of routine, preventative health care.

### 3.4. Education Access and Quality

Education access and quality is one of the SDOH domains that is most frequently mentioned in literature discussing SDOH and burn injuries. Educational access is easily reported, often requiring patients to respond with their highest level of completed education. An interesting observation is the global nature of much of the work being done to report educational status and access as a variable associated with the incidence of burn injuries. Many of the studies reporting this association are conducted in low- and middle-income countries; however, even in the United States, as previously mentioned, access to education is highly variable and plays a significant role in health outcomes. Many studies report on the patient or caretaker’s level of education being associated with burn risk, injury, or severity [38,43,45,77,78,79,80]. Low levels of education were also associated with a higher risk for suicide by burns [81], and better maternal education has been shown to be a protective factor of pediatric burns [38,82].

Fewer studies drew conclusions about the association between the level of education and outcomes for burn injuries. Meng et al. conducted a systematic review and determined that a low maternal education was associated with late complications of burns such as contractures [83]. Romanowski et al. demonstrated that higher levels of education were associated with reductions in the length of hospital stays and the risk of being discharged to a skilled nursing facility for elderly burn patients [52]. An analysis of the Life Impact Burn Recovery Evaluation dataset showed that level of education was correlated with social reintegration after burn injuries in patients younger than 30 years of age [84]. Even fewer studies analyze the impact of education quality on burn risk or outcomes, possibly due to the difficulty in measuring the quality of education across different countries and even US regions. Measures such as reading or math proficiency are means of assessing education quality; however, these data are not commonly collected in healthcare or health outcomes research [85].

### 3.5. Social and Community Context

Though the definition of this domain seems straightforward (“relationships and interactions with family, friends, co-workers, and community members” [7]), these relationships, and the consequences of them, can manifest in different ways. It may describe the relationship between a burn patient and their healthcare provider and how easily the two communicate. It may also describe how mental health challenges, such as anxiety and depression, influence a patient’s risk for self-harm via burns. Because of the range of this domain, studies that describe the association of the social and community context with burn injuries often do so with more specific aspects of social networks and communities. Psychiatric review of 19 pediatric burn patients and their families revealed that in 9 cases, significant emotional maladjustment was present in the child before the burn [86,87]. Kendall-Grove et al. surveyed parents of children admitted for burn treatment and found that in 36% of families who responded, at least one parent reported significant dysfunction such as substance abuse, incarceration, or a history of psychiatric illness [88]. The relationship between parent and child and the social support that this bond may facilitate can impact outcomes after burn injuries, including resilience. Jang et al. reported that in burn patients recovering in a rehabilitation center, self-esteem and family support showed statistically significant positive correlations with resilience [89].

Negative relationships can also be linked to burn injuries. Burns that are due to interpersonal violence (IPV) account for 1.1–10% of total global burn admissions [90,91]. In their retrospective review of the Burns Registry of Australia and New Zealand, Murphy et al. determined that half of IPV burns were due to family or domestic violence [92]. While there are many studies that describe the impact of burn injuries on relationships with the patient’s friends and families, there is a paucity of research that seeks to understand the converse. One study was able to report from their analysis of burn patients with greater than 60% TBSA that survivors of the injury were more likely than non-survivors to have social support, measured as whether a patient had family or friends present during an ICU stay [93]. The authors posit that in other fields, evidence strongly suggests that both the presence and quality of social support is impactful on patient outcomes, and they conclude that this is likely the case in burn injuries as well. They conclude by suggesting the need for further studies studying the influence of social support and connections pre-discharge [93]. Few other studies assess how relationships, both positive and negative, impact the risk for burn injuries or outcomes related to burns. Often, studies may use surrogate factors or behaviors that are the result of the social and community context of a patient, such as mental health, substance abuse, or incarceration. Future research should be conducted so that instead of drawing conclusions about the effect of social and community contexts from these downstream indicators, relationships are directly measured via tools such as the Multidimensional Scale of Perceived Social Support [94] and analyzed for their impact on burn patients.

### 3.6. Neighborhood and the Built Environment

The preventative nature of burn injuries is perhaps why the SDOH domain of the built environment is most reported on in the literature. Design modifications to buildings and homes have been a target of public health practitioners when focusing on how to prevent burn-related injuries and fatalities. This has culminated in efforts such as passing legislation to require residential heaters be set below a certain temperature to avoid scald burns [95] or requiring the installation of household smoke detectors and automatic sprinkler systems [96].

Studies have proven significant associations between certain aspects of the built environment on the risk of injury and mortality from burns, such as the study by Runyan et al., who described that mobile homes posed a higher risk of death if there was a fire, fatal fires were more likely to be caused by smoking, and that the presence of a person who was impaired by alcohol was associated with death in a fire [97]. Aside from just the structure and design of buildings and homes, the built environment also refers to other aspects such as the design of our cities, the amount of open space present in our neighborhoods, and transportation systems. Research that focuses more on the neighborhood aspect of this domain similarly proves that these factors are associated with the risk for who gets burned and how. In Baltimore, Maryland, Schachterle et al. found that proximity to vacant properties increased the risk for home fires: for every vacant structure within 10 m, the risk of fire in the home increased by 8% [98]. Poverty and rural location, two factors that are often related, was shown to have the strongest influence on burn risk in an elderly population based on a study conducted on data from the North Carolina Jaycee Burn Center [99]. The neighborhood in which a person resides is often a product of other SDOH, and a data analysis measure called the Area Deprivation Index (ADI) allows for a multifaceted evaluation of an area’s socioeconomic conditions [100]. Purcell et al. reported in their retrospective analysis of burn admissions in North Carolina that most patients admitted were grouped in the highest ADI, or the most disadvantaged quartile. They also found that patients in this ADI quartile had an increased risk of ≥20% for TBSA and inhalational injury than patients in the second-lowest ADI quartile [101]. As a greater emphasis shifts from risk factors to the social determinants of health, more studies will likely begin to look at the importance of these interwoven and related factors, such as the neighborhood and the built environment, in the burn patient population.

## 4. Viewing Burn Care through the Lens of Social Determinants

The complex, resource-intensive nature of burn care, the preventative and environmental characteristics of the causes of burns, and the existing evidence base showing that social and economic factors are associated with risks for burn injuries and poor outcomes after injuries all emphasize the need for burn health care providers to adopt the paradigm that the social determinants of health need to be central when treating and studying the patient population. While this narrative review has its limitations in that the potential bias of the authors exists, nonetheless, it demonstrates that the burn research and literature are beginning to espouse this change. In order to advocate for comprehensive, preventative action and create more supportive environments, the field of burn surgery should invest its time and resources into conducting research to acquire more specific data about the SDOH [5]; form partnerships with other fields and champions, such as community health workers and educators, to provide multidisciplinary care and prevention; and advocate for social change via community engagement and calls for legislative reform in order to transform the data about these SDOH into effective policy that protects patients [30,102]. While these goals are lofty and easier said than done, especially considering the demands on a physician’s time from both patients and administrations, who better to advocate for the burn patient than the healthcare teams that care for them? At the very least, burn providers should have a basic understanding of the SDOH and the literature that supports its influence on burn patients, and an idea of how to approach care through the lens of these determinants.

## Figures and Tables

**Table 1:** Overview of the social determinants of health domains and associated search terms with screened and accepted articles.

Database/SDH Domain	Search Terms	# Articles Returned	# Full Articles Screened	# Articles Kept
**Economic Stability**	(socioeconomic factors or socioeconomic influence) AND (burns or burn injury or burns trauma or major burns) (‘socioeconomic factors’/exp OR ‘socioeconomic factors’ OR ‘economic stability’/exp OR ‘economic stability’) AND (‘burn’/exp OR burn) (“socioeconomic factors”[MeSH Terms] OR „economic stability”[All Fields]) AND „burns”[Text Word]	5045	82	11
**Healthcare Access and Quality**	“healthcare access and quality” AND ( burns or burn injury or burns trauma or major burns) (‘health care access’/exp OR ‘health care access’) AND burn Healthcare access AND burn[Text Word]	446	63	12
**Education Access and Quality**	education access AND (burns or burn injury or burns trauma or major burns) education AND access AND ‘burn’ “Education”[Mesh] AND burn[Text Word]	1050	45	14
**Social and Community Context**	(social environment or social support or social relationships) AND burns or burn injury or burns trauma or major burns ‘social environment’ AND burn “Social Environment”[Mesh] AND burn[Text Word]	682	48	9
**Neighborhood and Built Environment**	built environment or neighborhood AND burns or burn injury or burns trauma or major burns (‘built environment’/exp OR ‘built environment’) AND (‘burn’/exp OR burn) “Environment Design”[Mesh] AND burn	90	22	5

## Data Availability

All studies reviewed in this paper have been appropriately cited and are publicly accessible.

## References

[1] Centers for Diseases Control and Prevention Social Determinants of Health: Know What Affects Health. https://www.cdc.gov/socialdeterminants/index.htm.

[2] McGinnis J.M., Williams-Russo P., Knickman J.R. (2002). The case for more active policy attention to health promotion. Health Aff..

[3] Sharma M., Pinto A.D., Kumagai A.K. (2018). Teaching the Social Determinants of Health: A Path to Equity or a Road to Nowhere?. Acad. Med..

[4] World Health Organization Social Determinants of Health. https://www.who.int/health-topics/social-determinants-of-health#tab=tab_1.

[5] Magnan S. (2017). Social Determinants of Health 101 for Health Care: Five Plus Five. NAM Perspect..

[6] Lewis J.H., Lage O.G., Grant B.K., Rajasekaran S.K., Gemeda M., Like R.C., Santen S., Dekhtyar M. (2020). Addressing the Social Determinants of Health in Undergraduate Medical Education Curricula: A Survey Report. Adv. Med. Educ. Pract..

[7] Healthy People 2030, U.S. Department of Health and Human Services, Office of Disease Prevention and Health Promotion. Social Determinants of Health. U.S. https://health.gov/healthypeople/objectives-and-data/social-determinants-health.

[8] Taylor L.A., Tan A.X., Coyle C.E., Ndumele C., Rogan E., Canavan M., Curry L.A., Bradley E.H. (2016). Leveraging the Social Determinants of Health: What Works?. PLoS ONE..

[9] World Health Organization Burns. https://www.who.int/news-room/fact-sheets/detail/burns.

[10] Hopkins S. (2006). Economic stability and health status: Evidence from East Asia before and after the 1990s economic crisis. Health Policy.

[11] Swain G.R. (2017). How does economic and social disadvantage affect health. Focus.

[12] Osborn R., Squires D., Doty M.M., Sarnak D.O., Schneider E.C. In New Survey of 11 Countries, U.S. Adults Still Struggle with Access to and Affordability of Health Care. The Commonwealth Fund. https://www.commonwealthfund.org/publications/journal-article/2016/nov/new-survey-11-countries-us-adults-still-struggle-access-and.

[13] Keisler-Starkey K., Bunch L.N. Health Insurance Coverage in the United States: 2020. United States Census Bureau. https://www.census.gov/library/publications/2021/demo/p60-274.html.

[14] Collins S.R., Gunja M.Z., Aboulafia G.N. U.S. Health Insurance Coverage in 2020: A Looming Crisis in Affordability. The Commonwealth Fund. https://www.commonwealthfund.org/publications/issue-briefs/2020/aug/looming-crisis-health-coverage-2020-biennial.

[15] Kitagawa E.M., Hauser P.M. (1973). Differential Mortality in the United States: A Study in Socioeconomic Epidemiology.

[16] Zajacova A., Lawrence E.M. (2018). The Relationship Between Education and Health: Reducing Disparities Through a Contextual Approach. Annu. Rev. Public Health.

[17] Bowlby J. (1969). Attachment and Loss.

[18] Berkman L.F., Glass T., Berkman L., Kawachi I. (2000). Social integration, social networks, social support, and health. Social Epidemiology.

[19] Hernandez L.M., Blazer D.G., Institute of Medicine (US) Committee on Assessing Interactions Among Social, Behavioral, and Genetic Factors in Health (2006). Chapter 2: The Impact of Social and Cultural Environment on Health. Genes, Behavior, and the Social Environment: Moving Beyond the Nature/Nurture Debate.

[20] Stansfeld S., Marmot M., Wilkinson R. (1999). Social support and social cohesion. Social Determinants of Health.

[21] Cohen S., Underwood L.G., Gottlieb B.H. (2000). Social Support Measurement and Intervention.

[22] Cassel J. (1976). The contribution of the social environment to host resistance: The fourth Wade Hampton Frost lecture. Am. J. Epidemiol..

[23] Centers for Disease Control and Prevention Impact of the Built Environment on Health. https://www.cdc.gov/nceh/publications/factsheets/impactofthebuiltenvironmentonhealth.pdf.

[24] Peters A., Pope C.A. (2002). Cardiopulmonary mortality and air pollution. Lancet.

[25] Sallis J., Glanz K. (2006). The role of built environments in physical activity, eating, and obesity in childhood. Future Child..

[26] Giskes K., van Lenthe F., Avendano-Pabon M., Brug J. (2011). A systematic review of environmental factors and obesogenic dietary intakes among adults: Are we getting closer to understanding obesogenic environments?. Obes. Rev..

[27] Glasgow Centre for Population Health The Built Environment and Health: An Evidence Review. https://www.gcph.co.uk/assets/0000/4174/BP_11_-_Built_environment_and_health_-_updated.pdf.

[28] Hollands G.J., Marteau T.M., Fletcher P.C. (2016). Non-conscious processes in changing health-related behaviour: A conceptual analysis and framework. Health Psychol. Rev..

[29] Andermann A., CLEAR Collaboration (2016). Taking action on the social determinants of health in clinical practice: A framework for health professionals. CMAJ.

[30] NEJM Catalyst Social Determinants of Health (SDOH). https://catalyst.nejm.org/doi/full/10.1056/CAT.17.0312.

[31] Healthy People Foundation Health Measures Archive. Office of Disease Prevention and Health Promotion. https://www.healthypeople.gov/2020/About-Healthy-People/Foundation-Health-Measures2020/About-Healthy-People/Foundation-Health-Measures/Archive.

[32] Gómez C.A., Kleinman D.V., Pronk N., Gordon G.L.W., Ochiai E., Blakey C., Johnson A., Brewer K.H. (2021). Addressing Health Equity and Social Determinants of Health Through Healthy People 2030. J. Public Health Manag. Pract..

[33] World Health Organization Health impact assessment. https://www.who.int/health-topics/health-impact-assessment#tab=tab_1.

[34] Forjuoh S.N. (2006). Burns in low- and middle-income countries: A review of available literature on descriptive epidemiology, risk factors, treatment, and prevention. Burns.

[35] Peck M.D., Kruger G.E., van der Merwe A.E., Godakumbura W., Ahuja R.B. (2008). Burns and fires from non-electric domestic appliances in low and middle income countries Part I. The scope of the problem. Burns.

[36] World Health Organization (2008). The Global Burden of Disease: 2004 Update. www.who.int/healthinfo/global_burden_disease/GBD_report_2004update_full.pdf.

[37] Alnababtah K., Khan S., Ashford R. (2016). Socio-demographic factors and the prevalence of burns in children: An overview of the literature. Paediatr. Int. Child Health..

[38] Edelman L.S. (2007). Social and economic factors associated with the risk of burn injury. Burns.

[39] Marsden N.J., Battle C.E., Combellack E.J., Sabra A., Morris K., Dickson W.A., Whitaker I.S., Evans P.A. (2016). The impact of socio-economic deprivation on burn injury: A nine-year retrospective study of 6441 patients. Burns.

[40] Istre G.R., McCoy M., Carlin D.K., McClain J. (2002). Residential fire related deaths and injuries among children: Fireplay, smoke alarms, and prevention. Inj. Prev..

[41] Forjuoh S.N., Guyer B., Smith G.S. (1995). Childhood burns in Ghana: Epidemiological characteristics and home-based treatment. Burns.

[42] Petridou E., Trichopoulos D., Mera E., Papadatos Y., Papazoglou K., Marantos A., Skondras C. (1998). Risk factors for childhood burn injuries: A case-control study from Greece. Burns.

[43] Daisy S., Mostaque A.K., Bari T.S., Khan A.R., Karim S., Quamruzzaman Q. (2001). Socioeconomic and cultural influence in the causation of burns in the urban children of Bangladesh. J. Burn. Care Rehabil..

[44] Karim K., Alam I., Ui-Hasan N., Khan A. (1975). The socio-economic factors of burn injuries in children. Burns.

[45] Delgado J., Ramírez-Cardich M.E., Gilman R.H., Lavarello R., Dahodwala N., Bazán A., Rodríguez V., Cama R.I., Tovar M., Lescano A. (2002). Risk factors for burns in children: Crowding, poverty, and poor maternal education. Inj. Prev..

[46] Forjuoh S.N. (1996). Burn repetitions in Ghanaian children: Prevalence, epidemiological characteristics and socioenvironmental factors. Burns.

[47] Padalko A., Cristall N., Gawaziuk J.P., Logsetty S. (2019). Social Complexity and Risk for Pediatric Burn Injury: A Systematic Review. J. Burn Care Res..

[48] Padalko A., Gawaziuk J., Chateau D., Sareen J., Logsetty S. (2020). Social Determinants Associated with Pediatric Burn Injury: A Population-Based, Case-Control Study. J. Burn Care Res..

[49] Argirova M., Arnautska E. (2020). Ethnic and social factors in toddlers with extensive burns in the Bulgarian paediatric population: Problems, dilemmas, solutions. Ann. Burn Fire Disasters.

[50] Mohammadzade M.J., Rarani M.A., Keyvanara M. (2020). Social and medical determinants of burn-related mortality in Isfahan, Iran. Arch. Trauma. Res..

[51] Karashchuk I.P., Solomon E.A., Greenhalgh D.G., Sen S., Palmieri T.L., Romanowski K.S. (2021). Follow-up after Burn Injury is Disturbingly Low and Linked with Social Factors. J. Burn Care Res..

[52] Romanowski K.S., Zhou Y., Eyck P.T., Baldea A., Gallagher J.J., Galet C., Liu Y.M. (2021). Racial And Socioeconomic Differences Affect Outcomes in Elderly Burn Patients. Burns.

[53] Titi N., van Niekerk A., Ahmed R. (2018). Child understandings of the causation of childhood burn injuries: Child activity, parental domestic demands, and impoverished settings. Child Care Health Dev..

[54] Dissanaike S., Ha D., Mitchell D., Larumbe E. (2017). Socioeconomic status, gender, and burn injury: A retrospective review. Am. J. Surg..

[55] American Psychological Association Social Deprivation. https://dictionary.apa.org/social-deprivation.

[56] Mistry R.M., Pasisi L., Chong S., Stewart J., She R.B. (2010). Socioeconomic deprivation and burns. Burns.

[57] Mendonca A.D., Da Silva A.F., Turner J., Sen A. (2004). The relation between social deprivation and incidence of burns in an A&E setting. Burns.

[58] Duncanson M., Woodward A., Reid P. (2002). Socioeconomic deprivation and fatal unintentional domestic fire incidents in New Zealand 1993–1998. Fire Saf. J..

[59] Kaufman M.S., Graham C.C., Lezotte D., Fauerbach J., Gabriel V., Engrav L., Esselman P. (2007). Burns as a result of assault: Associated risk factors, injury characteristics, and outcomes. J. Burn Care Res..

[60] Sethi J., Gawaziuk J.P., Cristall N., Logsetty S. (2018). The Relationship Between Income and Burn Incidence in Winnipeg, Manitoba, Canada: A Population Health Study. J. Burn Care Res..

[61] Locke J.A., Rossignol A.M., Boyle C.M., Burke J.F. (1986). Socioeconomic factors and burn rates in persons hospitalized for burns in Massachusetts. Public Health Rep..

[62] Rossignol A.M., Locke J.A., Burke J.F. (1989). Employment status and the frequency and causes of burn injuries in New England. J. Occup. Med..

[63] Locke J.A., Rossignol A.M., Burke J.F. (1990). Socioeconomic factors and the incidence of hospitalized burn injuries in New England counties, USA. Burns.

[64] American Burn Association Burn Incidence Fact Sheet. https://ameriburn.org/who-we-are/media/burn-incidence-fact-sheet/.

[65] Hop M.J., Polinder S., van der Vlies C.H., Middelkoop E., van Baar M.E. (2014). Costs of burn care: A systematic review. Wound Repair Regen..

[66] Farrell R.T., Gamelli R.L., Sinacore J. (2006). Analysis of functional outcomes in patients discharged from an acute burn center. J. Burn Care Res..

[67] Peluso H., Abougergi M.S., Caffrey J. (2018). Impact of primary payer status on outcomes among patients with burn injury: A nationwide analysis. Burns.

[68] Blumenthal D., Collins S.R., Fowler E. The Affordable Care Act at 10 Years: What’s the Effect on Health Care Coverage and Access?. The Commonwealth Fund..

[69] Dvorak J.E., Lester E.L.W., Maluso P.J., Tatebe L.C., Bokhari F. (2021). The Impact of the Affordable Care Act on Burn Outcomes. J. Burn Care Res..

[70] Dalton M.K., Riviello R., Kubasiak J.C., Sokas C.M., Osman S.Y., Jin G., Nitzschke S.L., Ortega G. (2021). Impact of the affordable care act’s medicaid expansion on burn outcomes and disposition. Burns.

[71] American Burn Association Burn Center Regional Map. http://ameriburn.org/public-resources/burn-center-regional-map/.

[72] Carmichael H., Wiktor A.J., McIntyre R.C., Lambert Wagner A., Velopulos C.G. (2019). Regional disparities in access to verified burn center care in the United States. J. Trauma Acute Care Surg..

[73] Klein M.B., Kramer C.B., Nelson J., Rivara F.P., Gibran N.S., Concannon T. (2009). Geographic Access to Burn Center Hospitals. JAMA.

[74] Klein M.B., Nathens A.B., Emerson D., Heimbach D.M., Gibran N.S. (2007). An analysis of the long-distance transport of burn patients to a regional burn center. J. Burn Care Res..

[75] Bodily N.E., Bruenderman E.H., Bhutiani N., The S., Schucht J.E., Bozeman M.C. (2021). The Effect of Transfer on Outcomes in Burns. J. Burn Care Res..

[76] Shaarbafchi Zadeh N., Mohammadi F., Amini Rarani M., Javadi M., Mohammadzade M., Yazdi-Feyzabadi V. (2019). How the service delivery works in the Iranian specialised burns hospitals? A qualitative approach. PLoS ONE.

[77] Adil S.O., Ibran E.A., Nisar N., Shafique K. (2016). Pattern of unintentional burns: A hospital-based study from Pakistan. Burns.

[78] Adil S.O., Nisar N., Ehmer-Al-Ibran Shafique K., Baig-Ansari N. (2016). Severity of burn and its related factors: A study from the developing country Pakistan. Burns.

[79] Chen X., Sun W., Wang J., Han D., Gao G., Yan D., Zhao X., Yao X., Wang L., Wang G. (2014). Epidemiology of bedside stove burns in a retrospective cohort of 5089 pediatric patients. Burns.

[80] Shi S., Yang H., Hui Y., Zhou X., Wang T., Luo Y., Xiang H., Shi X. (2016). Epidemiologic characteristics, knowledge and risk factors of unintentional burns in rural children in Zunyi, Southwest China. Sci. Rep..

[81] Lari A.R., Joghataei M.T., Adli Y.R., Zadeh Y.A., Alaghehbandan R. (2007). Epidemiology of suicide by burns in the province of Isfahan, Iran. J. Burn Care Res..

[82] Forjuoh S.N., Guyer B., Strobino D.M., Keyl P.M., Diener-West M., Smith G.S. (1995). Risk factors for childhood burns: A case-control study of Ghanaian children. J. Epidemiol. Community Health.

[83] Meng F., Zuo K.J., Amar-Zifkin A., Baird R., Cugno S., Poenaru D. (2020). Pediatric burn contractures in low- and lower middle-income countries: A systematic review of causes and factors affecting outcome. Burns.

[84] Schulz J.T., Shapiro G.D., Acton A., Fidler P., Marino M.E., Jette A., Schneider J.C., Kazis L.E., Ryan C.M. (2019). The Relationship of Level of Education to Social Reintegration after Burn Injury: A LIBRE Study. J. Burn Care Res..

[85] Roser M. Measuring Education: What Data is Available? Our World in Data. https://ourworldindata.org/measuring-education-what-data-is-available.

[86] Heath J. (2016). Depression: An Antecedent and Consequence of Burn-Injuries to Children. Austin J. Emerg. Crit. Care Med..

[87] Long R.T., Cope O. (1961). Emotional Problems of Burned Children. N. Engl. J. Med..

[88] Kendall-Grove K.J., Ehde D.M., Patterson D.R., Johnson V. (1998). Rates of dysfunction in parents of pediatric patients with burns. J. Burn Care Rehabil..

[89] Jang M.H., Park J., Chong M.K., Sok S.R. (2017). Factors Influencing Resilience of Burn Patients in South Korea. J. Nurs. Scholarsh..

[90] O’Halloran E., Duke J., Rea S., Wood F. (2013). In the media: Burns as a method of assault. Burns.

[91] Peck M.D. (2012). Epidemiology of burns throughout the World. Part II: Intentional burns in adults. Burns.

[92] Murphy L., Read D., Brennan M., Ward L., McDermott K. (2019). Burn injury as a result of interpersonal violence in the Northern Territory Top End. Burns.

[93] Muangman P., Sullivan S.R., Wiechman S., Bauer G., Honari S., Heimbach D.M., Engrav L.H., Gibran N.S. (2005). Social support correlates with survival in patients with massive burn injury. J. Burn Care Rehabil..

[94] Zimet G.D., Dahlem N.W., Zimet S.G., Farley G.K. (1988). The Multidimensional Scale of Perceived Social Support. J. Personal. Assess..

[95] Erdmann T.C., Feldman K.W., Rivara F.P., Heimbach D.M., Wall H.A. (1991). Tap water burn prevention: The effect of legislation. Pediatrics.

[96] Staunton C.E., Frumkin H., Dannenberg A.L., Doll L.S., Bonzo S.E., Sleet D.A., Mercy J.A. (2008). Changing the Built Environment to Prevent Injury. Handbook of Injury and Violence Prevention.

[97] Runyan C.W., Bangdiwala S.I., Linzer M.A., Sacks J.J., Butts J. (1992). Risk factors for fatal residential fires. N. Engl. J. Med..

[98] Schachterle S.E., Bishai D., Shields W., Stepnitz R., Gielen A.C. (2012). Proximity to vacant buildings is associated with increased fire risk in Baltimore, Maryland, homes. Inj. Prev..

[99] Hendrix L., Charles A., Buchholz V., Jones S., Cairns B. (2011). Influence of race and neighborhood on the risk for and outcomes of burns in the elderly in North Carolina. Burns.

[100] Maroko A.R., Doan T.M., Arno P.S., Hubel M., Yi S., Viola D. (2016). Integrating Social Determinants of Health With Treatment and Prevention: A New Tool to Assess Local Area Deprivation. Prev. Chronic. Dis..

[101] Purcell L.N., Bartley C., Purcell M.E., Cairns B.A., King B.T., Charles A. (2021). The effect of neighborhood Area Deprivation Index on residential burn injury severity. Burns.

[102] Braveman P., Egerter S., Williams D.R. (2011). The Social Determinants of Health: Coming of Age. Annu. Rev. Public Health.

